# Compliance with nice guidelines for management of depression in a community mental health team

**DOI:** 10.1192/bjo.2021.875

**Published:** 2021-06-18

**Authors:** Sophie Mellor, Shay-Anne Pantall, Lisa Brownell

**Affiliations:** 1 University of Birmingham, Medical School; 2 Birmingham and Solihull Mental Health NHS Foundation Trust

## Abstract

**Aims:**

To evaluate compliance within a Community Mental Health Team (CMHT) to the NICE guidelines for the management of depression.

**Background:**

Reducing the prevalence of depression continues to be a major public health challenge.

Given the complexity and recurrent nature of the condition, the NICE guideline CG90 is an invaluable resource to aid the effective management of depression. Here we present an audit of adherence to this guideline within a CMHT.

**Method:**

A retrospective electronic casenote review of all patients diagnosed with depression between January 2016 and October 2019 under the care of a Birmingham CMHT (n = 35), assessing key performance areas including: quality of assessment and coordinated care, risk assessment, choice of pharmacological and psychological treatment using the stepped care model and appropriate crisis resolution planning.

**Result:**

Key results include:

- The majority of patients were Caucasian (63%). Ages ranged from 27 to 69 (mean age 48 years old).
- Severity of disorder was typically moderate (46%) or severe (48%). Of those with a diagnosis of severe depression, 41% had associated psychotic symptoms.
- Psychiatric comorbidity was high (49%), of which generalised anxiety disorder was the most common (59%).
- Referrals were typically from primary care (77%). Approximately half (51%) had reported suicidal thoughts according to the referral.
- A quarter of patients (26%) were seen by CMHT within 8 weeks of referral; 20% of referrals however waited over 12 months before being assessed.
- Risk assessments were out of date for 71% of patients.
- 100% of patients had a crisis plan noted within their most recent clinic letter; however, none of these met the required standards.

Polypharmacy was common (60%), with 34% prescribed two antidepressants. Use of lithium augmentation was uncommon, with only one patient prescribed this. 43% were prescribed an antipsychotic; of which, 29% had appropriate physical health monitoring completed.

Over half of patients (60%) had been referred to psychology services; of these, 38% had either completed or were in ongoing treatment at the time of review.

**Conclusion:**

CMHTs manage the care of individuals with depression who have high levels of active symptoms and disability, psychiatric comorbidity, care requirements, and complex treatment plans. Pharmacological management was broadly in line with guidelines, and rates of referral to psychology were satisfactory. Risk assessment and crisis planning are clear areas in need of urgent attention in order to comply with guidelines and ensure patient safety.

